# A Fully Soft and Passive Assistive Device to Lower the Metabolic Cost of Sit-to-Stand

**DOI:** 10.3389/fbioe.2020.00966

**Published:** 2020-08-14

**Authors:** Hangil Lee, Seok Hee Kim, Hyung-Soon Park

**Affiliations:** ^1^Department of Mechanical Engineering, Korea Advanced Institute of Science and Technology (KAIST), Daejeon, South Korea; ^2^Exercise Physiology, Korea Advanced Institute of Science and Technology (KAIST), Daejeon, South Korea

**Keywords:** soft, passive, metabolic cost, assistance, wearable, X-tights, sit-to-stand

## Abstract

Various assistive devices like exoskeletons have been developed to aid the growing number of disabled people. Recent studies have started to explore using soft rather than rigid components to create lightweight and unobtrusive systems that can be more easily adopted by the general population. However, there is a tradeoff between compliance and power in these systems. We investigated the physiological benefits of using an inconspicuous, soft and passive assistive device which would avoid bulkiness, heaviness and user discomfort. We chose to assist the sit-to-stand (STS) maneuver because it is a common activity of daily living (ADL). STS is also recognized as one of the most challenging ADLs due to the high knee torque required, and the primary limiting factor is known to be knee extensor strength. Thus, the objective of this research was to develop and evaluate an unobtrusive knee assist wear called X-tights that could aid knee extension during STS using only soft and passive components. This was accomplished by routing elastic bands across the lower extremity. Thirty-one healthy participants performed STS tests with and without the X-tights, while metabolic cost and muscle activity were recorded. Metabolic power significantly decreased, by 3.2 ± 1.5% (*P* = 0.04), when utilizing the X-tights during the STS, while there was no statistically significant differences in muscle activity. The present work introduces a new soft and passive assist wear that can be worn inconspicuously under normal clothing, and we demonstrate promising results for the future development and integration of soft assistive technology for daily life.

## Introduction

As the population of elderly persons around the world grows, the number of people with disabilities in need of assistive devices is steadily increasing. Emerging technologies have drastically improved the capacity of assistive devices like exoskeletons to restore a measure of functionality for simple activities of daily living (ADL), while reducing energy costs. Exoskeletons traditionally rely on powerful actuators linked to rigid structural components to provide substantial force transfer (Zoss et al., 2006; Tsukahara et al., 2010; Young and Ferris, 2017). These hard robots are well suited for applications that require high power outputs like assisting paraplegics in clinical settings (Whitesides, 2018). However, rigid assistive devices may force users into unnatural movements that create undesirable biomechanics due to joint misalignment (Rossi et al., 2013; Zanotto et al., 2015) and are often not suitable for prolonged daily use, because of their weight, size, and battery life (Browning et al., 2007; Veale and Xie, 2016).

Recent studies have started to explore soft alternatives, to create assistive devices that can be inconspicuously worn in daily life, using lightweight and compliant materials (Panizzolo et al., 2016; Chung et al., 2018; Sridar et al., 2018). Instead of rigid and bulky structures, soft devices can conform to the body’s natural mechanics and minimize user discomfort. These soft systems have a serious disadvantage, however: their maximum transferable torques (and/or force) are limited by the soft materials. Soft actuation techniques using special property materials are presently limited both in the amount of power they can generate and by difficulties in manufacturing for large scale orthosis applications (Veale and Xie, 2016). Many soft devices still rely on hard actuators like electric motors or pneumatic pistons and batteries to power the soft components. But these rigid components re-introduce issues of excessive bulk and weight (Panizzolo et al., 2016; Schmidt et al., 2017; Chung et al., 2018; Sridar et al., 2018). Fully soft assistive devices have not yet been practically achieved because their inadequate power typically limits benefits from energy savings or muscle activity. The development of unobtrusive assistive wearables for daily living will require further research to resolve these shortcomings.

Our goal was to develop a fully soft and passive device that can be worn inconspicuously to assist one of the most common yet challenging ADLs for the elderly: the sit-to-stand (STS) maneuver (Riley et al., 1991). STS involves a sequential motion of rising to stand from a seated position, and performing it requires several joints to work in synergy, as well as substantial knee extensor strength.

An STS is considered to be successful if a person can rise out of a chair without losing balance. The importance of this STS cannot be overstated, as it is the precursor to other ADLs and essential to daily function. Impaired STS motions can lead to injuries from falling that may even be life threatening. Hence, the STS is often used as an index for independent living, and losing the strength to perform it can be debilitating.

In previous literature, the primary limiting factor in the STS sequence has been identified as knee extensor strength (Hughes et al., 1996). Knee extensor strength is key to straightening the legs and positioning the body’s center of mass (COM) over the base of support (Riley et al., 1997). The peak knee torque generated during the STS occurs during the initial knee extension phase, when the thigh is horizontally positioned and the effective lever arm of the COM is greatest (Yoshioka et al., 2014). The loss of knee extensor strength needed to overcome this peak torque has been linked to various factors; notably, muscle atrophy has been shown to reduce elderly subjects’ isokinetic knee flexor strength by 15% per decade (Virgina et al., 2001; Petrella, 2004).

In addition, the biomechanical stress of the STS may aggravate symptoms of knee diseases like osteoarthritis. One study suggested that a reduction in muscle co-contractions was linked to a reduction in knee compartmental joint pressure which led to decreased pain in the knee for osteoarthritis patients (Ramsey et al., 2007). Providing knee extension assistance may lower muscle co-contractions and subsequently reduce the pain and aggravation of osteoarthritis (Ramsey et al., 2007). Therefore, bolstering knee extensor strength for STS may have multiple benefits, including improved metabolic effort, muscle activity, and joint loading, which can carry over to better quality of life and the prevention of knee injury.

In this paper, we present an entirely soft and passive knee extension assist wear and evaluate its physiological benefits. No hard components or actuators were used to create the compliant device, which can be worn inconspicuously under normal clothing and has minimal weight. The system is simple, affordable and can be easily integrated into active devices. Studies have shown that older adults often prefer simpler assistive technology that are as inconspicuous as possible to avoid advertising their need for assistive devices (Jung and Ludden, 2019). Our work focuses on bridging the gap between basic engineering and its widespread application for the future integration of assistive technology into daily life.

To provide passive knee extension assistance, this study developed an optimized tendon routing method using elastic bands inspired by the anatomical design of human knee extensors for effective force transfer across the lower body. The effectiveness of the unobtrusive assist wear was evaluated by measuring the change in metabolic cost during STS tests. The test participants’ muscle activity was also recorded using surface electromyography (EMG), and a qualitative assessment was made by surveying the participants’ perceived exertion after each trial.

## Materials and Methods

### Knee Assist Wear Design

**FIGURE 1:**
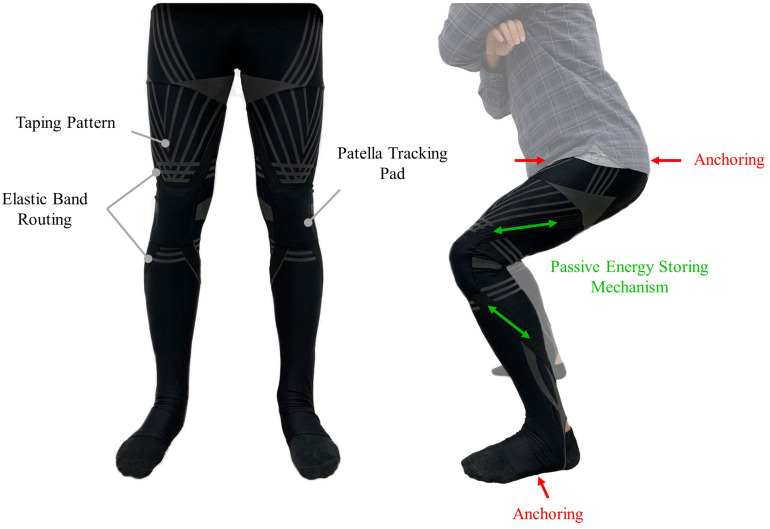
The soft and passive assist wear, the X-tights, designed in this study. The passive energy storing mechanism consists of four elastic bands that originate at the patella tracking pad covering the knee. The bands store energy as the user sits down and then release it when the user rises to stand, to assist the STS. Anchoring is achieved by a waistband on the hip bone and an extended leg hole that wraps underneath the foot.

The fundamental design concept of the assist wear was to incorporate a passive energy storing mechanism mimicking knee extensors. Elastic bands were selected as the energy storing component because of their light weight and compliant characteristics. The proposed approach routed the elastic bands across the front of the knee so that they would stretch and store energy when sitting and then return it when standing, to assist the STS (Figure 1). The stiffness of the elastic band was selected so that it would store adequate energy, but without completely overcoming the gravitational force used for sitting. In this way the energy storing phase during stand-to-sit would not create more work for the user but instead aid the user in descending slowly. A patella tracking pad made of silicon was used to avoid concentrated pressure on the patella that would cause discomfort.

**FIGURE 2:**
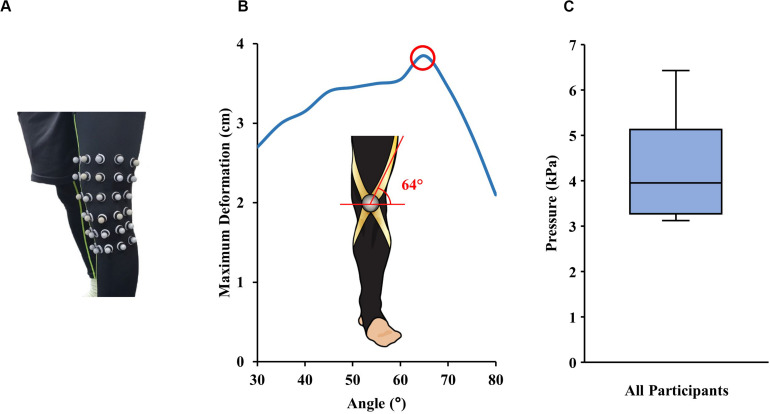
**(A)** A grid of 30 motion capture markers were spread across the knee to create a mesh surface to calculate the path of greatest deformation during STS. **(B)** The optimal band routing path was determined to be 64 degrees, by plotting skin deformation along the routing angle during knee flexion. **(C)** A boxplot of the maximum tolerable pressure around the waist for four participants.

A band routing analysis was developed to determine the route that would allow the band to stretch maximally across the front of the knee when sitting down, to store the greatest amount of elastic energy. Assuming the elastic band would follow the deformation in the skin, the optimal route of the band was defined as the direction of greatest skin deformation across the knee when flexed from fully straightened to 90 degrees. To find the optimal route, a mesh of the knee surface was created using motion capture (VICON motion systems, Oxford, United Kingdom) data from 30 reflective markers spread across the front of the knee (Figure 2A). The route of greatest deformation was calculated to travel through the center of the knee at 64 degrees off the horizontal (Figure 2B).

The elastic bands formed an X shape over the knee along the determined route. However, the overlapping center of the elastic bands applied concentrated pressure to the patella when sitting. This also misaligned the patella position and inhibited effective force transfer. To address these problems, a patella tracking silicon pad was inserted at the crossing point of the bands to eliminate painful pressure points. A stiff silicon material (KE-1300T, Shin-Etsu Chemical Co., Ltd., Nagoya, Japan) that would not stretch before the bands prevented loss of force transfer.

**FIGURE 3:**
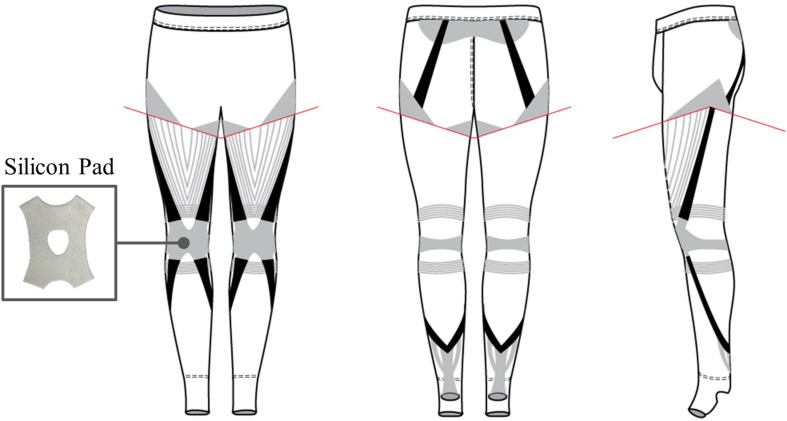
Schematic of the X-tights design with the elastic bands in black and the taping patterns in gray. The red lines at the thigh indicate a stitch that anchors the quadriceps taping. The horizontal taping around the knee section and the silicon patella tracking pad were used to prevent misalignment of the knee joint during dynamic motion.

The elastic bands were stitched into polyester tights to be worn over the lower body, to create adequate anchoring without the need for hard components. The top of the tights is anchored at the hipbones with an elastic waistband, and the bottom is extended through the leg hole to wrap under the foot (Figure 3). The tops of the bands extend to the waist and wrap around the backside to provide hip extension assist as well. The bottom portions of the bands are wrapped below the calf instead of the bottom of the tights to prevent interference with the ankle joint. The waistband and foot wrap prevent slippage caused by the tension of the stretched elastic bands. The friction required to stop slippage around the waistband can require significant tension and potential discomfort, so band stiffness was limited to prevent the assist wear slipping, or excessive discomfort to the user.

The suitable band stiffness, which is correlated to the maximum band tension, was estimated based on the maximum allowable anchoring force. To quantify this, the maximum tolerable pressure around the waist was experimentally found. This was done by gradually tightening an elastic band wrapped around four participant’s (age: 27.5 ± 3.8; BMI: 22.3 ± 0.93; mean ± SD) waist until they reported the pressure was too uncomfortable for prolonged wear. Measurements were repeated on a separate day to reduce influence of the pants worn. Using the known stiffness properties of the band, the pressure on the waist was approximated using the formula,

(1) $$\text{P}=\frac{2\,\pi \,\Delta \,x}{\text{LW}}$$

where Δx is stretched length of the band, L is circumference of the waist, and W is the width of the band. The average tolerable pressure was 4.2 kPa (Figure 2C) although the range varied between 3.1 to 6.4 kPa as each participant’s preference for wearing tight clothes differed (Yandell et al., 2020). Thus, a conservative threshold of 3.1 kPa was determined for the maximum allowable waist pressure which was then used to calculate the maximum tolerable tension force before slipping. These estimations were conservative because they assumed a cylindrical body and did not account for the boney hip and glute anatomy that naturally help prevent pants from sliding down. A maximum elastic band stiffness of 470 N/m was determined based on the waistband threshold pressure and coefficient of friction to prevent slipping and ensure comfortable wear.

Then, elastic taping was integrated into the tights to provide additional assistive benefits besides those from the elastic bands. Sports taping has commonly been used to induce muscle tension by physiotherapists (Nosaka et al., 2008) and has lately been incorporated into sportswear for similar benefits. To bolster knee extensor strength, taping patterns mimicking the quadriceps femoris were added to the design (Figure 3). Additionally, patterns mimicking the soleus and gastrocnemius were included to help the band anchor down to the heel. The total weight of the X-tights was 300 grams.

### Experimental Protocol

Thirty-one healthy participants (age: 43.8 ± 10.2; BMI: 21.6 ± 2.2; mean ± SD) who had no history of knee injury or heart conditions were gathered for this study. All participants provided written informed consent, and the study protocol was approved by the Institutional Review Board at KAIST. An STS protocol was designed to measure the changes in metabolic and muscle effort when wearing the X-tights. A modified version of the STS test (Bohannon, 1995) was used, in which a participant would stand from a seated position and then sit down again at a rate of 20 repetitions per minute for a total of 5 min. We will henceforth use STS to refer to this full cycle of STS-to-sit.

**FIGURE 4:**
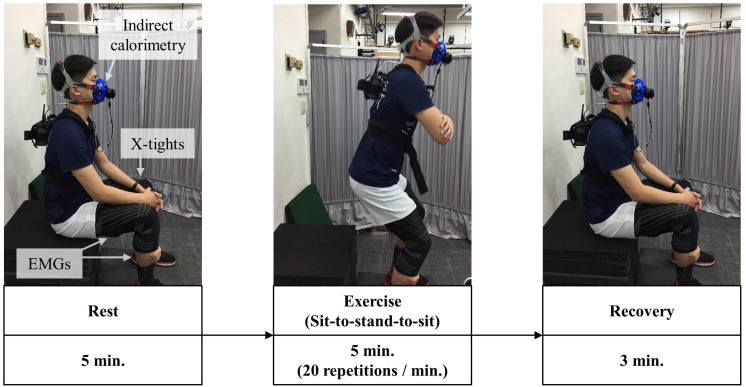
The experimental sequence protocol of a participant with the X-tights condition is shown. The participant began with 5 min of rest, followed by 5 min of exercise, and finished with 3 min of recovery.

A metronome kept the participant moving at a consistent pace, and if the participant felt they could no longer physically continue or could not follow the tempo for two consecutive repetitions, the trial was stopped. Verbal cues were given to ensure the participant fully straightened their knees and hips when standing and did not fall behind tempo. The seat height was adjusted so the participant’s knee flexion would be at 90 degrees when seated, and they were instructed to fold their arms during the exercise. The exercise was prefaced by a 5-min rest phase and followed by a 3-min recovery phase to record the participants’ resting and recovery metabolism (Figure 4). Participants performed the task with no brace and with the X-tights 3 days apart in random order. No brace was the control condition in which the participant performed the task without any extra assist wear. X-tights was the experimental condition utilizing the developed assist wear. A minimum of 3 days of rest between trials was required to allow the participant to fully recover from any fatigue (Bishop et al., 2008), and exercise was not permitted between trials. After each trial, the participant’s self-assessed perceived exertion was surveyed using the Borg rating of perceived exertion (RPE) that ranges from 6 (no exertion) to 20 (maximal exertion) (Borg, 1982).

### Data Analysis

Metabolic cost was assessed by indirect calorimetry using the Cosmed K5 (Cosmed, Rome, Italy). The two main metabolic parameters analyzed were the metabolic power calculated by the Brockway equation (Brockway, 1987) and respiratory exchange ratio (RER), which is VCO_2_ produced divided by VO_2_ consumed. The former is a common standard used to evaluate the rate of metabolic energy consumption, and the latter is used as an indicator of fatigue (Balady et al., 2010). Net metabolic power (Galiana et al., 2016) was found by subtracting the average metabolic power of the rest phase from the exercise phase which effectively removed the baseline metabolism of the participant, isolating the effects from the exercise. The average reduction in metabolic power and the RER of the last 2 min of the exercise phase when steady state had been reached (Bassett et al., 1997) were analyzed for percent reduction between the two conditions:

(2) $$\%\mathrm{Reduction}=\frac{{\text{x}}_{\text{nobrace}}-{\text{x}}_{\text{tights}}}{{\text{x}}_{\text{nobrace}}}\times 100$$

Thus, a positive value indicated a reduction or improvement when wearing the X-tights. Percentages were calculated for individual participants before calculating the inter-participant mean and standard error of the mean (SEM). The Paired *t*-test was used to test for statistical significance between the two conditions, and Shapiro-Wilk W. test was used to confirm the normality assumption beforehand.

Additional metabolic parameters, the anaerobic threshold and the rate of change of RER during exercise, were also analyzed. The anaerobic threshold reflects the point in time when oxygen supply to the muscle cannot meet the demand and lactate starts to accumulate in muscles. This parameter is also used as an assessment of cardiovascular health and can reflect the intensity of exercise for an individual (Wasserman, 1986). The anaerobic threshold was estimated non-invasively by evaluating the VO2 when RER has stabilized over 1.0, and it has been shown to correlate well with anaerobic threshold values derived from blood lactate measurements (Solberg et al., 2005). The rate of change of RER was calculated from the slope of a linear regression fit to the RER data during the exercise phase. This value reflects the rate of fatigue accumulation during exercise.

Muscle activity was measured using the Trigno Wireless EMGs System (Delsys Inc., Boston, MA, United States). Six EMGs were attached to the following muscles on each leg: rectus femoris (RF), vastus lateralis (VL), biceps femoris (BF), semitendinosus (ST), tibialis anterior (TA), and lateral gastrocnemius (LG). Each EMG sensor was placed in a small foam cushion and bound to the leg with wrapping tape to reduce artifacts from inertia while recording the dynamic movements. This also helped the sensors stay in place when the tights were worn over them. The bulkiness of the sensors around the shank, however, made it difficult for participants to wear the tights, so a small hole was cut in the X-tights at the location of the tibialis anterior sensor. If the sensors shifted when the participant put on the tights, they were readjusted to the appropriate locations. The maximum volumetric contractions (MVC) of each muscle was recorded prior to each trial (Halaki and Gi, 2012) and used to normalize the experimental data. All of the MVC values were measured in the initial sitting posture with the knee at 90 degrees of flexion while sitting on the seat. The EMG data was filtered by a Butterworth high pass filter, rectified, and then filtered by a low pass filter to form a linear envelope as recommended by past literature (Potvin and Brown, 2004). Ten STS cycles for each condition were used to compute the mean and standard deviation of the normalized EMG linear envelope. The left and right leg signals were averaged to produce six linear envelopes per condition.

The co-contraction of the thigh and shank muscles was also examined. Co-contraction refers to the combined activity of agonist and antagonist muscles around the same joint and is also referred to as agonist and antagonist co-activation (Banks et al., 2017; Latash, 2018). Co-contraction of the thigh and shank muscles was observed by plotting the overlapping regions of the EMG linear envelopes for the corresponding agonist and antagonist muscle pairs. The RF and BF were used for the thigh muscles, and the TA and LG were used for the shank muscles.

## Results

**FIGURE 5:**
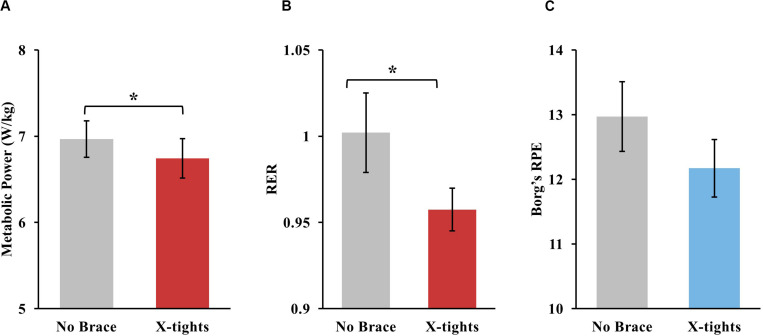
The average **(A)** metabolic power, **(B)** RER, and **(C)** RPE for the no brace and X-tights conditions, with SEM bars. The asterisk (*) signifies statistical significance between the two conditions.

There was an overall reduction in metabolic power and RER when wearing the X-tights. The metabolic power of 20 out of 31 participants was reduced when wearing the tights, and the overall reduction was 3.2 ± 1.5% (mean ± SEM) (Figure 5A). The average metabolic power of the no brace and X-tights conditions were 6.97 ± 1.17 W/kg and 6.74 ± 1.27 W/kg, respectively. The RER results were similar for 21 out of 31 participants, who showed a reduction in fatigue when wearing the tights, for an overall reduction of 3.6 ± 1.5% (Figure 5B). The average RER of the no brace and X-tights conditions were 1.00 ± 0.13 and 0.96 ± 0.07, respectively. The average RPE reported by participants after each trial was 12.97 ± 0.54 and 12.17 ± 0.45 for the no brace and X-tights conditions, respectively (Figure 5C).

Statistical analysis indicated that the reduction in metabolic power and RER from wearing the X-tights was significant. The Paired *t*-test showed statistical significance for the reduction in metabolic power (*P* = 0.04) and RER (*P* = 0.02) for the trial conditions of no brace or X-tights. The difference in RPE did not pass the normality test and was not tested for significance.

The additional metabolic parameters also showed slight improvement when wearing the X-tights. Anaerobic threshold was not available for every trial because not all subjects reached an RER of 1.0. To evaluate this, subjects were categorized into improved, worsened, or not available groups. Improved was when the subject’s anaerobic threshold was higher when wearing the tights or if the subject reached an RER of 1.0 only during no brace condition. Worsened was vice versa and not available referred to both trials not reaching an RER of 1.0. The anaerobic threshold of 10 participants improved, 9 worsened, and 12 were not available. Most participants did not reach the anaerobic threshold during the exercise. The average reduction of rate of change of RER was 11.02 ± 5.45% when wearing the X-tights compared to no brace condition. This was expected and correlated with the reduced RER.

**FIGURE 6:**
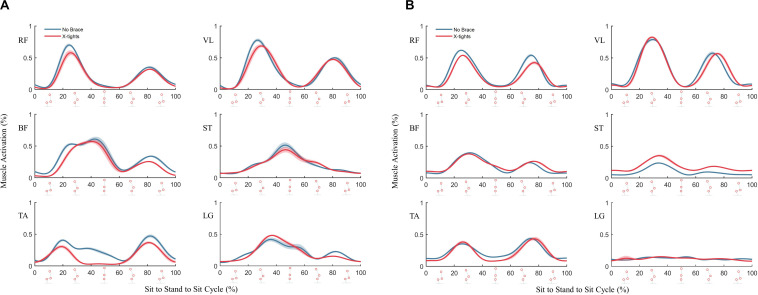
Normalized EMG linear envelope for the STS cycle for **(A)** a participant who showed reduction in muscle activity and **(B)** a participant who showed similar activation patterns. The blue and red solid lines show means of 10 cycles for the no brace and X-tights conditions, respectively, and the shaded regions represent the standard error.

The results of muscle activity obtained from the EMGs were less conclusive than the metabolic results. The normalized EMG linear envelopes showed high variability between participants (Supplementary Table S1). The normalized linear envelopes of a participant A, who showed reduction in muscle activation when wearing the X-tights (Figure 6A), and a participant B, who showed similar activation patterns between conditions (Figure 6B), illustrate these inter-participant differences. Although the activation patterns of the knee extensors, RF and VL, were consistent across participants, the other muscle groups showed varying activation patterns. For instance, participant B showed little LG activation compared to participant A. The differences in muscle recruitment strategy likely occurred because only the starting posture for the STS was controlled. The Paired *t*-test was conducted for the muscle activity reduction, but no main effects were found for the six muscles. Unlike the metabolic results, there was no discernable change in muscle activity between the no brace and X-tights conditions.

**FIGURE 7:**
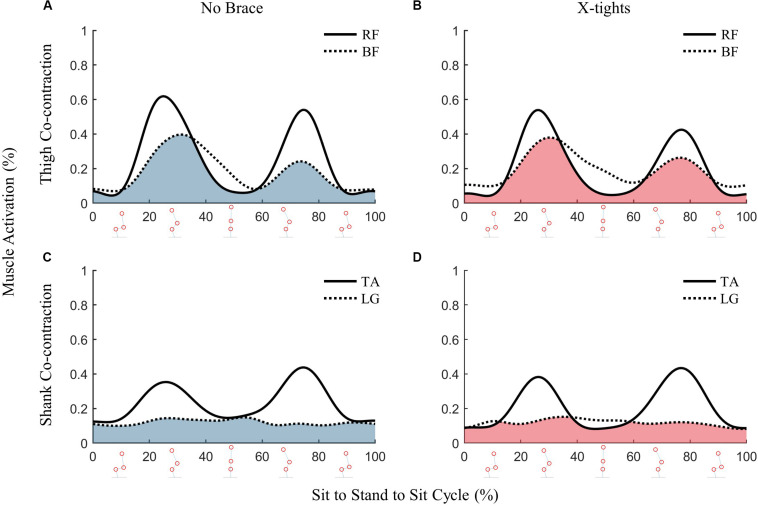
The co-contractions for the thigh muscles and shanks muscles of participant B are shaded in blue and red for the no brace and X-tights conditions, respectively. The linear envelopes of the thigh muscles, RF and BF, are plotted in the top row. The linear envelopes of the shank muscles, TA and LG, are plotted in the bottom row. **(A)** The thigh co-contractions for the no brace conditions and **(B)** thigh co-contractions for the X-tights show similarity in magnitude and shape. **(C)** The shank co-contractions for the no brace conditions, and **(D)** shank co-contractions for the X-tights were much lower as participant B had minimal LG activation.

Analysis of the thigh and shank co-contractions also showed no discernable patterns between the two conditions. The co-contraction plots for participant B illustrate nearly identical patterns for both conditions (Figure 7). High levels of co-contraction for thigh muscles occur during the transition from STS and stand-to-sit. This corresponds with biomechanically demanding postures that require the most stabilization due to the body’s displaced center of gravity. The co-contraction for the shank were much lower as participant B had minimal LG activation.

## Discussion

In this study, we investigated the potential metabolic benefits an inconspicuous, soft and passive assistive device could provide. No actuators or rigid components were used for the simple and compliant wearable, which could be worn unobtrusively under normal clothing. The metabolic cost was significantly reduced when performing the STS with the X-tights. The average reductions in metabolic power and RER were 3.2 ± 1.5% and 3.6 ± 1.5%, respectively; the X-tights not only reduced energy expenditure when performing the STS, but also reduced fatigue when the exercise was continuously performed. The variability in the metabolic cost reduction across participants may be due to factors like the varying fit of the X-tights, and individual neuromuscular adaptation to wearing the assist wear. The additional metabolic parameters evaluated are not commonly analyzed in exoskeleton studies but were included to illustrate a more comprehensive picture of the metabolic benefits. The reduction in rate of change of RER suggest that the X-tights slows the onset of fatigue, but the anaerobic threshold did not show definitive results because the task was not intensive enough for most participants. Monitoring the anaerobic threshold would have been more informative for a higher intensity task. The slight reduction in RPE suggests that users also perceived the X-tights as being beneficial by reducing exertion during the STS. These results demonstrate the feasibility of developing entirely soft and passive assistive devices that can be worn inconspicuously with normal clothes for daily use. The X-tights were optimized to assist the STS because it is one of the most challenging ADL, but benefits could be applied to similar tasks by incorporating the same design principles.

To the best of our knowledge, there are no other fully soft and passive devices that have been tested with STS, but we can draw comparisons to similar soft or passive assistive devices that have been shown to reduce metabolic cost for different activities. A study on an untethered soft exosuit for loaded walking measured a 14.9% reduction in metabolic power (Lee et al., 2018). This device demonstrated substantial effectiveness for a soft system, but it relied on an actuation and battery system that in total, with the suit, weighed 9 kg. A passive ankle exoskeleton using hard components reduced the metabolic cost of walking by 7.2% with a total weight of 1 kg (Collins et al., 2015). This exoskeleton was completely passive and vastly reduced weight by eliminating any actuators and batteries, but the rigid structure would be more difficult to wear under normal clothing. Both these devices are innovative examples of effective soft or passive systems.

The X-tights demonstrated a relatively lower reduction in metabolic cost, but offered advantages of minimal bulk and a total weight of only 0.3 kg. Traditional knee sleeves offer similar benefits of being lighter and simpler to use, making them a popular option for the general population with knee problems. However, they are often limited in anchoring force and prone to slipping given the difficulties of anchoring around the soft tissue of the thigh (Park et al., 2020). The X-tights utilized the hard components of the human body, the hipbone and the heels, to increase resistance to slipping without relying on rigid elements. This was an advantage of using the tights design rather than the traditional sleeve type design. To our knowledge, past studies on the benefits of soft and passive knee orthoses have been limited to subjective improvements and functional parameters (Mohd Sharif et al., 2017), and our study is the first to evaluate the metabolic benefits for the STS.

Although the X-tights’ effect on metabolic cost was observed in the experiment, the change in biological knee power from the passive assist mechanism during the STS was difficult to measure directly without invasive procedures. To provide more insight on the biomechanical benefits of the X-tights, the maximum assistive torque from the device and change in biological knee joint power were estimated using an STS simulation. A three-segment body model based on kinematic data was created to compute the inverse dynamics during the STS, while the X-tights were modeled as a set of passive springs attached to the knee joint. A detailed description of the simulation is included in the Supplementary Material. The maximum assistive torque at the knee was 0.03 Nm/kg for the selected band stiffness parameters, which corresponded to an 8.4% (0.08 W/kg) reduction in peak positive knee joint power during the standing phase, and a 9.1% (0.07 W/kg) reduction in peak negative knee joint power during the sitting phase (Supplementary Figure S1A).

Normally, storing energy requires additional external work, but here, the passive assist mechanism stores energy while helping the body oppose gravity, and then reduces the negative power for the stand to sit transfer (Supplementary Figure S1B). Although the simulation indicated a reduction in joint loading, there was no change in muscle co-contraction around the knee to suggest reduced compartmental joint pressure or risk of OA. An elastic band with higher stiffness would have stored more energy to induce a greater change in muscle activity, but would also have required greater anchoring forces. Furthermore, we ensured that the bands were not exceedingly stiff as to overcome the gravitational force, otherwise the user would exert additional energy and risk losing balance during the STS. The X-tights did not likely worsen the user’s balance because the muscle co-contraction around the ankle, which was shown to increase in response to postural instability (Melzer et al., 2001), remained unchanged.

This passive assist mechanism can also be incorporated into a hybrid actuated system to enhance effectiveness with minimal additional weight and complexity. The Myosuit is an example of a soft wearable device that combines passive and active components to assist hip and knee extension (Schmidt et al., 2017; Haufe et al., 2019). The active components were shown to reduce knee power by 35% during the STS, but the passive elements were not evaluated. A study from the Zelik lab presented a passive biomechanically assistive garment that reduced low back activation during lifting tasks using elastic bands that can be worn under normal clothing (Lamers et al., 2018). They also suggested working toward quasi-passive modules that can selectively activate passive assist mechanisms. The XoSoft project has developed a soft modular biomimetic exoskeleton that effectively utilizes these principles (Di Natali et al., 2019). A suit with quasi-passive structures to control passive modules for varying tasks may be a lightweight alternative to a fully active exoskeleton that can be more readily adopted by society. Our study demonstrated a band routing technique to optimize the benefits that a fully soft and passive assist mechanism alone can give, and future works can combine multiple quasi-passive modules to assist different ADLs.

Contrary to our expectations, no significant reductions in muscle activity were observed for any conditions despite a decrease in metabolic cost. Interestingly, a study done on a soft exosuit at Harvard similarly showed that a substantial reduction in muscle activity did not necessarily follow a significant reduction in metabolic cost (Panizzolo et al., 2016). They suggested that changes in the functional properties of the muscles rather than activation might have led to a reduced workload. Furthermore, the precise relationship between metabolic cost and muscle activity is still unclear, especially because EMG measurements are not directly indicative of muscle force production (Türker and Sözen, 2013; Vigotsky et al., 2018). Many factors that may have been altered by wearing the X-tights, such as muscle fascicle length, muscle geometry, and passive structural components, can also contribute to force production and cannot be identified with surface EMGs alone (Mesin et al., 2006; Massó et al., 2010; Farris et al., 2013). The present study was not equipped to analyze these physiological parameters. Future work in the area of human-exoskeleton adaptation and its effect on muscle activation will be important for the integration of assistive devices for the general population.

There were several limitations in the conducted study. There were only two sizes per gender, so the fit of the tights may have differed slightly between participants according to variations in physique. The taping on some of the X-tights started ripping after repeated testing, but the main assistive bands did not show signs of wearing. The number of trials may have been inadequate to show a statistical difference in muscle activity. There may also have been uncertainty in the EMGs when wearing the X-tights, due to the compression of the tights over the devices. Although the EMGs were put in cushions and wrapped, the pressure from the tights may have altered signals during dynamic motion in a manner that was not accounted for by the isometric MVCs. Holes could not be cut for every EMG, however, because this would alter the overall effect the X-tights would have on the participant. Other factors such as a training effect from the first trials or a change in diet may have affected performance between days; testing was done at similar times of the day with randomized trial orders to minimize these uncertainties.

The novelty of this work is the development and evaluation of an inconspicuous, soft and passive assist wear that stores and releases energy during the STS without any additional power supply. Our study focused on developing the practical aspect of technology that can be adopted by the general population. While there is a need to pursue highly advanced technology, there is also merit in investigating the application and evaluation of simple affordable devices. Presently, there is a void in research on fully soft and passive assistive devices because the limited power they can provide. Nonetheless, developing and evaluating these devices will be a crucial stepping point in the widespread integration of wearable technology by the general public.

In conclusion, the newly developed X-tights demonstrated the feasibility and merits of soft and passive assistive devices by reducing metabolic cost while being unobtrusive enough to wear under normal clothing. We minimized the size and weight of our device by optimizing an elastic band assist routing that stores energy from gravity for knee extension assistance during the STS. The same design principles outlined in this study can be applied to develop simple passive assistive mechanisms for other ADLs that can also be incorporated into existing active systems. With the growing number of disabilities, further work in the field of inconspicuous assistive devices can provide practical solutions that can be adopted by the general population and greatly improve the quality of daily life.

## Data Availability Statement

The raw data supporting the conclusions of this article will be made available by the authors, without undue reservation.

## Ethics Statement

The studies involving human participants were reviewed and approved by Institutional Review Board, Korea Advanced Institute of Science and Technology. The patients/participants provided their written informed consent to participate in this study. Written informed consent was obtained from the individual(s) for the publication of any potentially identifiable images or data included in this article.

## Author Contributions

HL and H-SP contributed to conception of the X-tights and developed the assist wear and drafted and revised the article. HL, SK, and H-SP designed the experimental protocol and facilitated the experiments. H-SP supervised this study. All authors contributed to the article and approved the submitted version.

## Conflict of Interest

HL and H-SP are inventors of the patent application (KR10-2018-0090759, pending), filed on August 3rd, 2018, for the proposed knee extension assist wear. The remaining author declares that the research was conducted in the absence of any commercial or financial relationships that could be construed as a potential conflict of interest.
