# Supplementary figures and images for: A Fully Soft and Passive Assistive Device to Lower the Metabolic Cost of Sit-to-Stand
Source: Front Bioeng Biotechnol. 2020 Aug 14;8:966. doi: 10.3389/fbioe.2020.00966 (PMC7456876; doi:10.3389/fbioe.2020.00966)

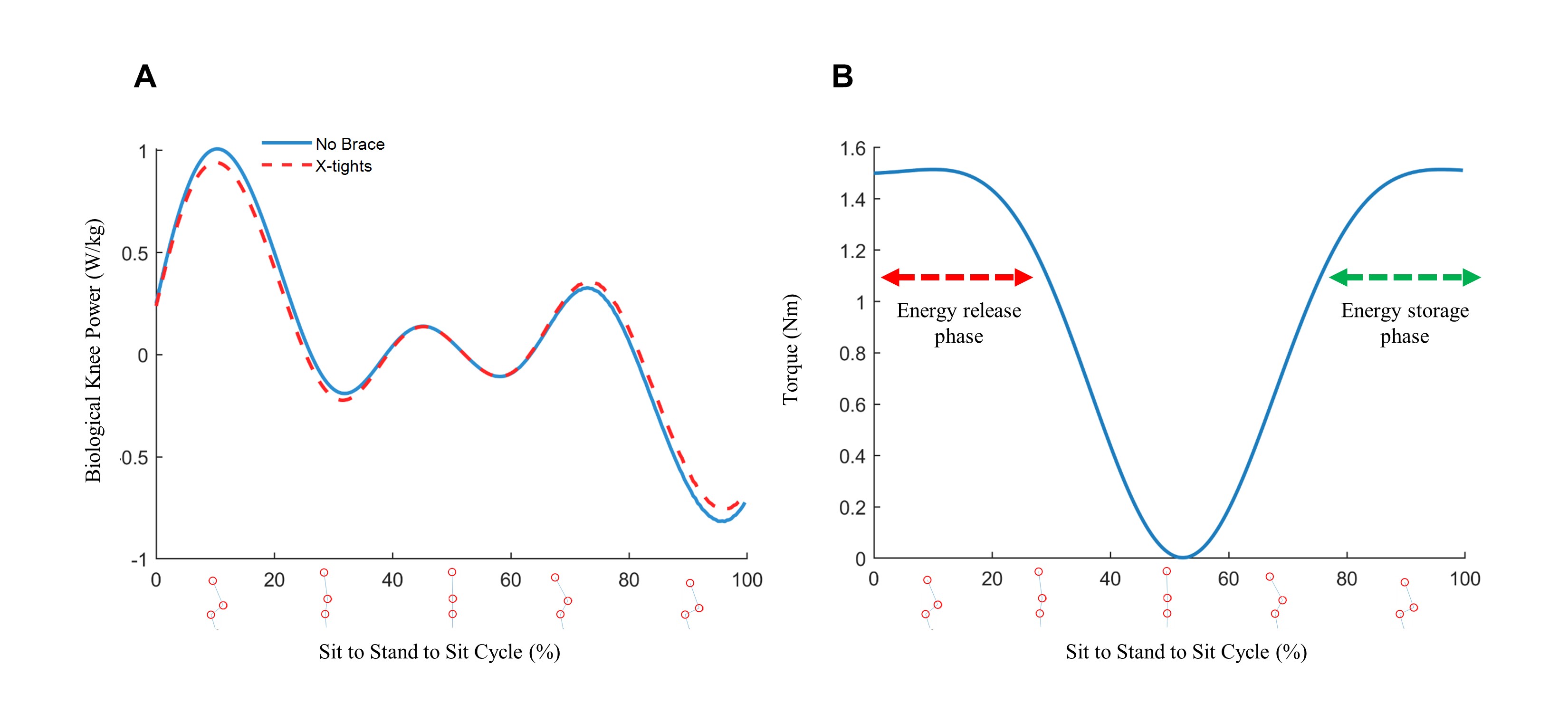

Supplement: Supplementary file 1 [file Image_1.jpg]
