# Supplementary material for: A Fully Soft and Passive Assistive Device to Lower the Metabolic Cost of Sit-to-Stand
Source: Front Bioeng Biotechnol. 2020 Aug 14;8:966. doi: 10.3389/fbioe.2020.00966 (PMC7456876; doi:10.3389/fbioe.2020.00966)
Supplement: Supplementary file 2 [file Table_1.docx]

Supplementary Material

# Supplementary Data

## Sit-to-Stand Simulation

The maximum assistive torque and changes in biological power at the knee from the X-tights was estimated using a sit-to-stand (STS) simulation. The simulation used input kinematic data of the sit-to-stand-to-sit and the elastic bands’ stretch to calculate the load at the ankle, knee, and hip joints. Based on past research, the STS was modeled using a three-segment body of the shank, thigh and HAT (head, arms, and trunk) with torque actuators at every joint in the sagittal plane (Musić, Kamnik, and Munih 2008; Hemami and Jaswa 1978; Yoshioka et al. 2007; Matthew et al. 2016; Sibella et al. 2003). Anthropometric parameters like body segment length, relative mass, and inertia were based on Leva’s parameters (Leva 1996). Motion data used to compute the inverse dynamics were recorded from motion capture of the STS, and the dynamics of the model were calculated using the Newton-Euler equations of motion. The biological knee power was found by multiplying the knee moment with the angular velocity. The input kinematic data from three healthy male subjects (age: 24 ± 1; mean ± SD) was measured as they performed the STS at a rate of 20 repetitions per minute off a seat adjusted to make knee flexion 90 degrees when sitting. Markers were attached to the spinous process of C7, sacroiliac joint, and bilaterally on the acromion, anterior superior iliac spine, epicondylus lateralis, lateral malleolus, and fifth metatarsal head. An additional 15 markers were attached to the elastic band portion of the X-tights to track the band’s stretch during movement.

The four elastic bands forming an X over the knee on the X-tights were modeled as four springs to simulate the passive energy storing mechanism. The elasticity from the tights and taping material were measured independently and modeled as an additional spring. Each spring was anchored at the center of the knee and stretched along the thigh or shank during knee flexion. The band stretch was modeled as a function of the angular knee displacement based on the motion capture data. These functions combined with the band stiffness parameter were applied to the three-segment model as external forces and torques during the inverse dynamics computations. The force functions for the top and bottom bands were applied along directional vectors pointing toward the knee joint along the thigh and shank segment, respectively.

# Supplementary Figures and Tables

## Supplementary Table

| Muscle | EMG Reduction (%) |
| --- | --- |
| RF | 6.3 ± 1.8 |
| VL | -0.6 ± 2.7 |
| BF | -0.4 ± 2.3 |
| ST | 6.4 ± 1.0 |
| TA | 9.8 ± 1.0 |
| LG | 6.9 ± 1.0 |

**Supplementary Table 1.** A table of the inter-participant mean and SEM EMG reduction when wearing the X-tights during the STS. RF, rectus femoris; VL, vastus lateralis; BF, biceps femoris; ST, semitendinosus; TA, tibialis anterior; LG, lateral gastrocnemius.

## Supplementary Figure


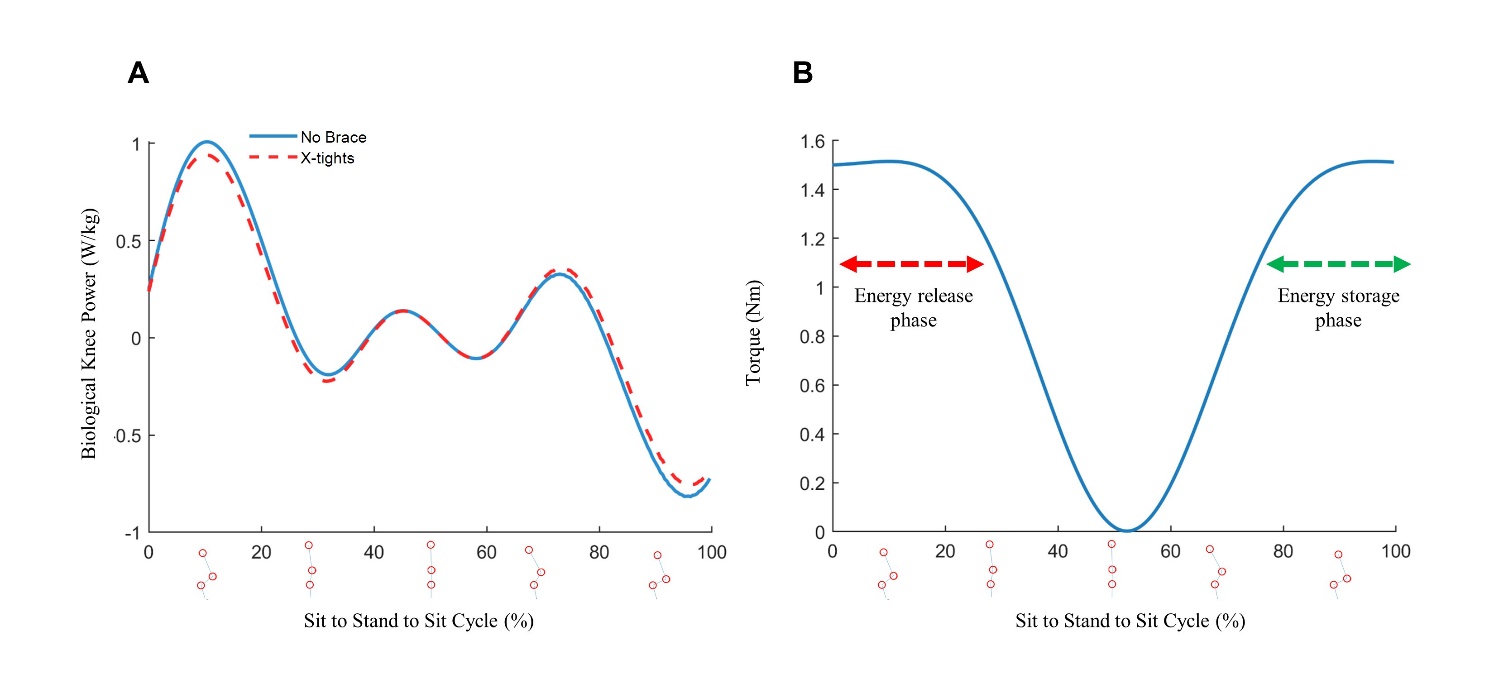


**Supplementary Figure 1.** (A) The biological knee power during the STS cycle for the no brace and X-tights conditions are shown, with the corresponding STS sequence pictured below each plot. (B)The assistive torque profile from the elastic band during the STS cycle. The energy storage phase matches the stand-to-sit and the energy release phase matches with the sit-to-stand.

**References**

Hemami, H., and Vijay C. Jaswa. 1978. “On a Three-Link Model of the Dynamics of Standing Up and Sitting Down.” *IEEE Transactions on Systems, Man and Cybernetics* 8 (2): 115–20. https://doi.org/10.1109/TSMC.1978.4309909.

Leva, Paolo. 1996. “Adjustments to Zatsiorsky-Seluyanov’s Segment Inertia Parameters.” *Journal of Biomechanics* 29 (9): 1223–30. https://doi.org/10.1016/0021-9290(95)00178-6.

Matthew, Robert Peter, Victor Shia, Gentiane Venture, and Ruzena Bajcsy. 2016. “Generating Physically Realistic Kinematic and Dynamic Models from Small Data Sets: An Application for Sit-to-Stand Actions.” *Proceedings of the Annual International Conference of the IEEE Engineering in Medicine and Biology Society, EMBS* 2016-Octob: 2173–78. https://doi.org/10.1109/EMBC.2016.7591160.

Musić, Josip, Roman Kamnik, and Marko Munih. 2008. “Model Based Inertial Sensing of Human Body Motion Kinematics in Sit-to-Stand Movement.” *Simulation Modelling Practice and Theory* 16 (8): 933–44. https://doi.org/10.1016/j.simpat.2008.05.005.

Sibella, F., M. Galli, M. Romei, A. Montesano, and M. Crivellini. 2003. “Biomechanical Analysis of Sit-to-Stand Movement in Normal and Obese Subjects.” *Clinical Biomechanics* 18 (8): 745–50. https://doi.org/10.1016/S0268-0033(03)00144-X.

Yoshioka, Shinsuke, Akinori Nagano, Ryutaro Himeno, and Senshi Fukashiro. 2007. “Computation of the Kinematics and the Minimum Peak Joint Moments of Sit-to-Stand Movements.” *BioMedical Engineering Online* 6: 1–14. https://doi.org/10.1186/1475-925X-6-26.
